# Pushing the limits of PLA by exploring the power of MWCNTs in enhancing thermal, mechanical properties, and weathering resistance

**DOI:** 10.1038/s41598-023-43660-3

**Published:** 2023-10-03

**Authors:** Mohammed M. Younus, Hamdy M. Naguib, Mohamed Fekry, Moataz A. Elsawy

**Affiliations:** 1 Special Processes Lab, Processes Development Division Egyptian Petroleum Research Institute, EPRI, Nasr City, Cairo 11727 Egypt; 2 Department of Petroleum Applications, Egyptian Petroleum Research Institute, Nasr City, Cairo 11727 Egypt; 3 Polymer Laboratory, Petrochemical Department, Egyptian Petroleum Research Institute, Naser City, Cairo 11727 Egypt

**Keywords:** Environmental social sciences, Materials science

## Abstract

The present study focuses on enhancing the mechanical, thermal, and degradation behavior of polylactic acid (PLA) by adding carbon nanotubes (CNTs) with different concentrations of 0.5, 1, 3, and 5%. The CNTs were prepared using catalytic chemical vapor deposition, and the prepared PLA/CNTs nanocomposite films were characterized using techniques such as FT-IR, Raman spectroscopy, TGA, SEM, and XRD. The distinct diffraction patterns of multi-walled carbon nanotubes (MWCNTs) at 2θ angles of 25.7° and 42.7° were no longer observed in the prepared nanocomposites, indicating uniform dispersion of MWCNTs within the PLA matrix. The presence of MWCNTs enhanced the crystallinity of PLA as the CNT loading increased. Mechanical tests demonstrated that incorporating CNTs positively influenced the elongation at the break while decreasing the ultimate tensile strength of PLA. The PLA-3%CNTs composition exhibited the highest elongation at break (51.8%) but the lowest tensile strength (64 MPa). Moreover, thermal gravimetric analysis confirmed that the prepared nanocomposites exhibited greater thermal stability than pure PLA. Among the nanocomposites, PLA-5% CNTs exhibited the highest thermal stability. Furthermore, the nanocomposites demonstrated reduced surface degradation in accelerated weathering tests, with a more pronounced resilience to UV radiation and moisture-induced deterioration observed in PLA-3% CNTs.

## Introduction

Polylactic acid (PLA) is a biodegradable thermoplastic polymer that has gained attention in biomedical and environmentally friendly fields due to its renewability, biocompatibility, and good processability. However, its intrinsic brittleness limits its practical applications. Despite efforts to increase its strength, modulus, and toughness, these properties have not been improved concurrently^1^. PLA is produced from renewable agricultural resources via fermentation and polymerization, making it an attractive sustainable alternative to traditional plastics as environmental pollution and fossil fuel scarcity become more pressing issues^2^. Reduced PLA production costs have expanded its end-use, including healthcare materials, packaging films, bottles, textile fibers, and high-performance engineering polymers for vehicle parts, demonstrating the significant potential for prospective applications as a high-performance bio-based plastic^3–8^. However, the significant degradation issue during the product's lifetime remains challenging for tougher engineering applications. Several hydrolytic degradation studies have been conducted on PLA to mimic its degradation in natural media, such as soil, compost, and the human body, at various temperatures^9^.

To enhance PLA’s degradability, researchers have investigated modifying its microstructure or blending it with other polymers, additives such as chitosan and plasticizers, and inorganic fillers^10–12^. Nevertheless, no material has yet been developed to monitor degradation and structural safety throughout the product's lifetime, reducing inspection and maintenance expenses. Carbon nanotubes (CNTs) are particularly promising fillers due to improving the mechanical properties and atomic structures of the final composites^13,14^. Researchers have extensively explored PLA nanocomposites reinforced with carbon nanotubes, which typically reduce the PLA matrix's elongation at the break while enhancing its tensile strength, modulus, and toughness^15,16^. The mechanical properties of CNT-filled nanocomposites are primarily influenced by interfacial adhesion and carbon nanotube dispersion. Surface functionalization is believed to enhance the affinity between PLA and carbon nanotubes by strengthening the interfacial interaction between carboxyl-functionalized carbon nanotubes and PLA. CNTs, composed of carbon and visualized as a single sheet of graphite rolled to form a seamless cylinder, have remarkable mechanical, physical, electrical, and other properties that make them promising candidates for polymer nanocomposites^17^.

This study aims to introduce a novel approach for enhancing the mechanical, thermal, dynamic mechanical, and degradation properties of polylactic acid (PLA) through the fabrication of nanocomposites using varying concentrations of homemade carbon nanotubes (CNTs). By forming a circuitous channel, CNTs effectively hinder the penetration of water molecules into the polymer matrix, thereby evaluating the hydrophobicity through contact angle measurements. The study employs ultrasonic homogenizers to achieve a homogeneous distribution of CNTs within the PLA matrix and evaluates the crystallinity of the resulting nanocomposites. The research focuses on addressing the brittleness of PLA while maintaining its environmental sustainability and biocompatibility. The findings contribute to the development of environmentally friendly packaging materials and assess the resistance of these materials to deterioration caused by exposure to moisture, heat, and UV radiation. The proposed methodology can revolutionize PLA applications in healthcare, packaging, textiles, and high-performance engineering. The study's outcomes provide valuable insights for advancing intelligent materials, replacing non-biodegradable plastics, and positively impacting the environment and human health.

## Materials and experiments

### Materials

Polylactic acid was obtained from Nature Works Ingeo LLC (Japan), specific gravity 1.24 g/cc, melt flow 10–30 g/10 min at load 2.16 kg at temperature 190 °C. Chloroform (99.0–99.4%) was obtained from Sigma-Aldrich. All the solutions were prepared using distilled water. All the chemicals were used as received and were of analytical grade. Cobalt nitrate hexahydrate (AG, Sigma-Aldrich) (Co(NO_3_)_2_·6H_2_O, < 98%), was used as a metal precursor for the preparation of supported catalysts. Deionized water, heavy Magnesium oxide MgO ≥ 98% (Carlo Erba reagents, GmbH, Germany) was used as the main support for catalysts.

### Preparation of carbon nanotubes

#### Preparation of the catalysts

To create the Co/MgO catalysts with a weight ratio of 50%, the required quantity of cobalt nitrate hexahydrate was dissolved in sufficient amounts of deionized water. The resulting solution was then added to the MgO support and thoroughly mixed to obtain a uniform mixture. This mixture was gently evaporated under continuous stirring at a temperature of 90 °C. The pastes obtained after evaporation were dried for a complete night at 120 °C, crushed into fine powders, and calcined for four hours at a temperature of 600 °C.

#### Synthesis of carbon nanotubes

The method used to create MWCNTs at EPRI (Egyptian Petroleum Research Institute) involved the Catalytic Chemical Vapor Deposition (CCVD) technique, utilizing a horizontal fixed-bed flow quartz reactor with ambient pressure. A dispersion of approximately 0.5 g of freshly calcined catalyst was placed on a quartz tube at the reactor's center. The catalyst underwent deoxidization as a recognized pre-treatment, where the temperature was raised to 700 °C under a hydrogen gas flow of 50 sccm and maintained at this temperature for 2 h. Afterward, 30 sccm of nitrogen (99.99%) was introduced, and the temperature was maintained for 5 min. For a 4 h process, a combination of 20 sccm of natural gas and 30 sccm of nitrogen was used. The flow of gases was regulated by digital mass flow controllers^18,19^.

### Preparation of PLA and its nanocomposite films with carbon nanotubes

PLA and its nanocomposite films with various amounts (0.5, 1, 3 and 5%) of CNTs have been prepared by using the solution casting method. Initially, PLA was dissolved in an appropriate amount of chloroform. Then the desired amount of CNTs was dispersed in PLA solution by sonicator, the sonication process occurred using inert metal vibrating probes from the titanium alloy (Ti-6Al-4V) submerged directly into the mixture for 30 min at a temperature 50 °C with 60% amplitude. The films were cut according to standard specifications for other tests.

### Characterization techniques

#### Fourier transform infrared spectroscopy and Raman spectra

Using an attenuated total reflection-Fourier transform infrared spectrophotometer (ATR-FTIR, Bruker Optik Gmbh Ettlingen, Germany), in the 4000–400 cm^−1^ range, the chemical structures were verified. Raman spectra were obtained using a (senterra)-Bruker, Germany at room temperature in small glass tubes by using 785 nm laser source.

#### X-Ray diffraction spectroscopy (XRD)

The XRD was investigated by a modern PAN analytical diffractometer, Xpert PRO model. Cu K-Alpha radiation (1.54060 Å) was utilized with 2θ ranging from 4.01° to 79.99° and a scanning rate of 0.02°/step at room temperature.

#### Ultrasonic processors or homogenizers

The sonication process occurred by using VCX-500 and VCX-750 ultrasonic processors.

#### Field emission scanning electron microscope FE‑SEM

Field emission scanning electron microscopy (FE-SEM) is a widely used technique to study the surface morphology of materials. For this study, we used the FEI Quanta FEG 250, manufactured by ZEISS. The procedure involves placing the sample in the FE-SEM chamber and scanning it with a focused electron beam. The electrons interact with the sample surface, producing secondary electrons that are detected by a detector to form the image.

#### Transmission electron microscopy (TEM)

TEM is a crucial tool for materials scientists due to its high-resolution imaging and microstructural analysis capabilities. Our JEOL JEM-200CX model from Japan is used for the procedure. The process involves creating thin samples of the material of interest on a carbon-coated copper grid, placing it in the TEM chamber, and exposing it to a high-energy electron beam. The resulting image is captured by a digital camera or projected onto a fluorescent screen, allowing for further analysis of characteristics like grain size, crystal structure, and defect density. TEM's exceptional imaging ability allows for a deeper understanding of materials at the nanoscale.

#### Particle size

The particle size distributions was measured with Malvern Zeta sizer nano series (NANO-ZS) HT using dynamic light scattering.

#### Thermal properties

The thermogravimetric sample analysis was achieved using SDT Q600 V20.9 Build 20 thermal gravimetric. Approximately 10 mg of the sample was placed in an alumina crucible, and the test was carried out from room temperature to 700 °C utilizing a heating rate of 10 °C/min.

#### Mechanical properties

The tensile test of samples was carried out with (Zwick/Roell/Z010). The standard test method (ASTM D 638) achieved the tensile test. The used load cell is 10 kN with a uniform rate speed of 500 mm min^−1^, at climate chamber 25 ± 2 °C**.** The prepared sample films were injection molded into standard dumbbell-shaped specimens. At least three specimens of each composition were tested, and the average values were reported.

#### Dynamic mechanical analysis

The mechanical behavior of the prepared PLA-CNT nanocomposites was investigated by a dynamic mechanical analyzer (DMA) using Triton Technology-TTDMA according to ASTM D 4065. The specimens’ sheets, with 25 mm length and 10 mm width, were heated from room temperature to 80 °C with 3 °C/min heating rate using bending mode.

#### Degradation studies

The degradation behavior of PLA and its nanocomposites with CNTs was evaluated using an accelerated weathering test. A QUV cyclic UV endurance device (120 V, 60-Hz model by QPanel Company, United States) with eight fluorescent UVA 340 lights (UV range 365–295 nm) was used. The test boards were exposed in the QUV chamber for 320 h as recommended in ASTM G154-00. The weight loss percentage of the prepared films was determined after treatment. The composite films were subjected to UV accelerated weathering conditions for 100 h at 95% humidity and 60 °C, following ASTM G154 protocol.

## Results and discussions

### FT-IR spectroscopy

**Figure 1:**
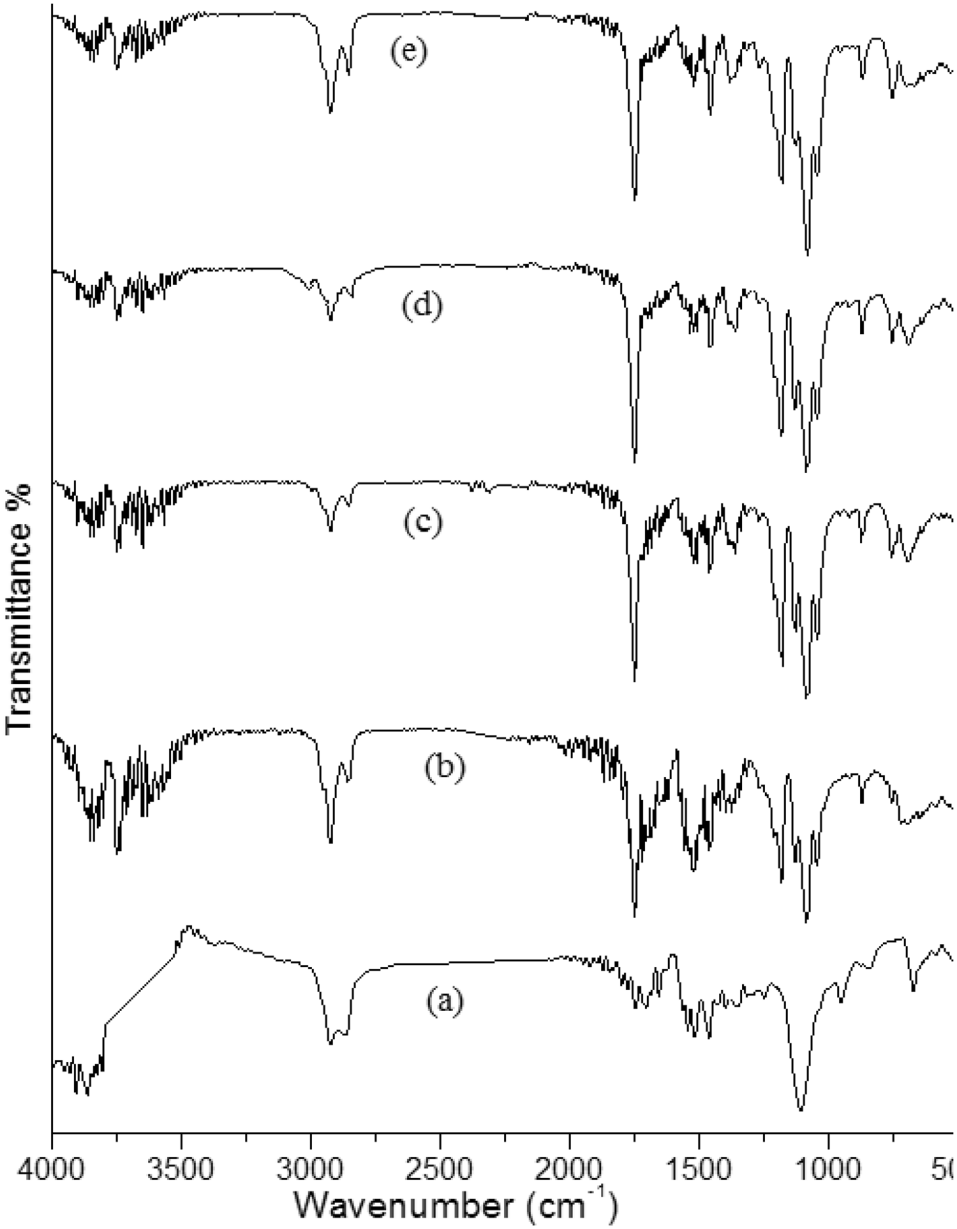
FT-IR spectra of (**a**) CNTs, (**b**) PLA, (**c**) PLA-0. 5% CNTs, (**d**) PLA-1% CNTs and (**e**) PLA-3.0% CNTs.

The Fourier transform infrared (FT-IR) spectra of carbon nanotubes (CNTs), polylactic acid (PLA), and various PLA/CNTs nanocomposites were obtained and are presented in Fig. 1. The pristine CNTs demonstrate characteristic absorption peaks at approximately 3440 cm^−1^, which can be attributed to the stretching vibration of hydroxyl groups. This peak may arise from atmospheric moisture that is bound to the CNTs. Furthermore, an absorption peak at 2924 cm^−1^ is observed, which corresponds to the stretching vibrations of the CH_2_ group. The characteristic peaks of CNTs are also observed at 1119 cm^−1^ (C–O) and 1600–1384 cm^−1^ (aromatic ring). The vibration band at 1627 cm^−1^ is attributed to the stretching mode of the benzenoid (υC=C) in the CNTs backbone, indicating the graphite structure of CNTs. Moreover, the characteristic peaks at 650 cm^−1^ correspond to the stretching vibrations of Co=O and Mg=O as catalysts^20^.

Pure PLA demonstrates three characteristic absorption bands at 3647, 1770, and 956–1100 cm^−1^, which correspond to the stretching vibrations of –OH, –C=O ester, and –C–O groups of PLA. The vibration modes at 2831–2993 and 1360–1445 cm^−1^correspond to the stretching and bending vibrations of CH_3_ and CH groups of PLA^21^. In the case of the PLA/CNTs nanocomposites, all the characteristic vibration bands corresponding to CNTs and PLA are present.

### Raman spectroscopy

**Figure 2:**
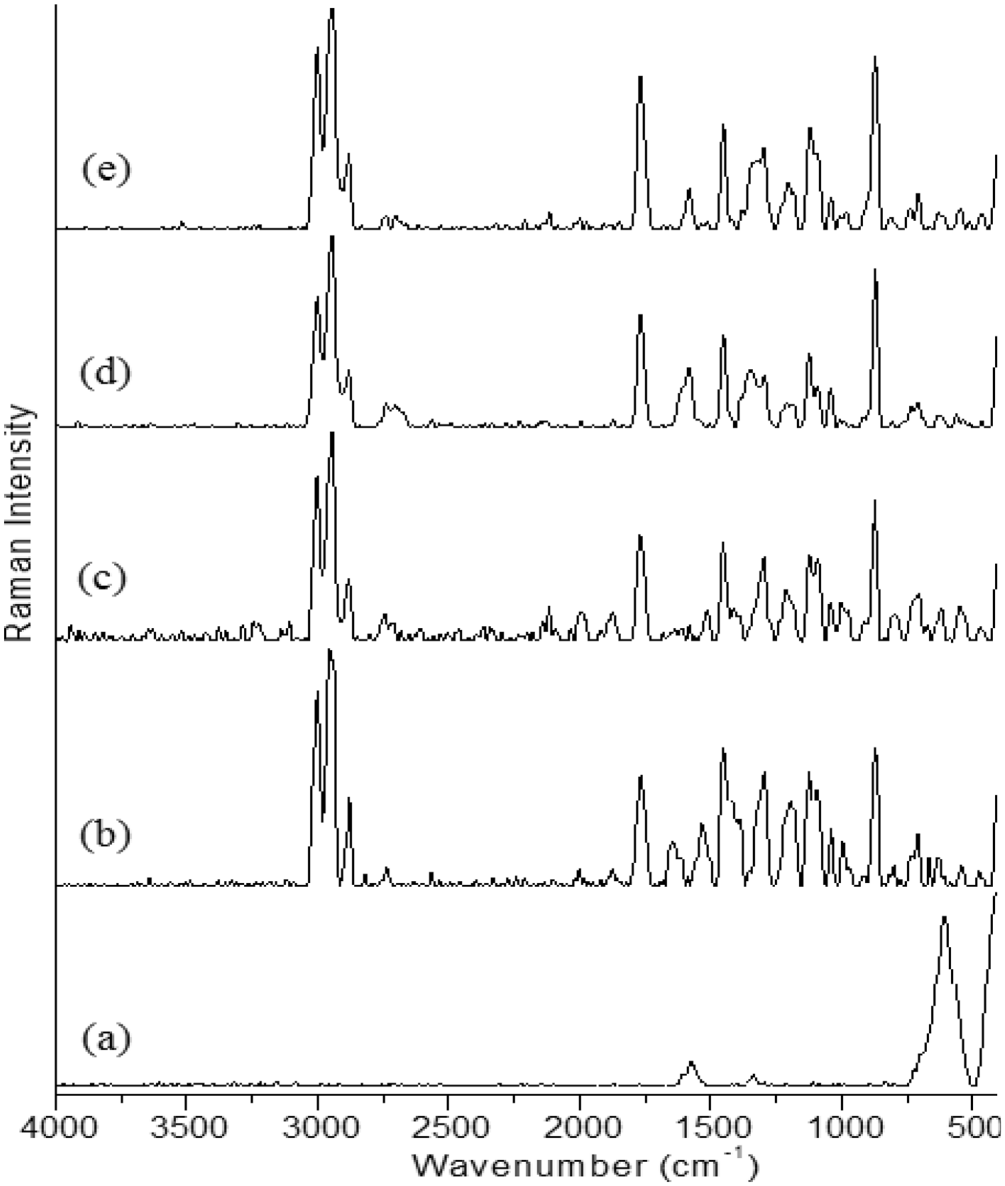
Raman Spectra of (**a**) CNTs, (**b**) PLA, (**c**) PLA-0. 5% CNTs, (**d**) PLA-1% CNTs and (**e**) PLA-3.0% CNTs.

The Raman spectra of CNTs, PLA, PLA-0.5% CNTs, PLA-1% CNTs, and PLA-3.0% CNTs are presented in Fig. 2. Raman spectroscopy is a widely used technique to characterize carbon nanostructures. The C–C bond vibrations of carbon nanotubes are known to undergo strain under various stresses such as tensile, compressive, and shear, resulting in changes in the Raman spectra. Both individual carbon nanotubes and nanotube-polymer composites have exhibited peak position and intensity changes due to stress. Raman spectroscopy assessed the degree of graphitization and crystallinity of the generated CNTs from the natural gas breakdown on Co/MgO catalysts. Two prominent bands, the D- and G-bands, were identified in all Raman spectra. The D-band observed at 1350 cm^−1^ is attributed to wall disorder or amorphous carbon formation outside the nanotubes. On the other hand, the G-band observed at 1580 cm^−1^ is responsible for the graphitization level in CNTs. The D- and G- intensity bands are commonly used to evaluate the quality of carbon nanotubes. The high intensity of the G-band compared to the D-band in the current operating conditions indicates that the CNTs are heavily graphitized, while the higher intensity of the D-band suggests a decrease in the crystallinity and purity of the produced carbon nanomaterials. The I_D_/I_G_ ratio of the D- and G-bands is indicates of the crystalline arrangement of CNTs. An I_D_/I_G_ value greater than one denotes a high degree of structural disorder in the carbon nanotubes produced on catalysts, while an I_D_/I_G_ value less than one signifies an increase in the degree of graphitization of the carbon deposition. The D- and G- properties of MWCNTs provide evidence of their presence in the composites. The D- and G-bands of the MWCNT/PLA composites exhibit a positional upshift compared to pure MWCNTs, with higher-frequency shifts seen particularly in the G-band. The decrease in nanotube-nanotube interactions resulting from the disentanglement and dispersion of CNT bundles in the polymer matrix is linked to this shift.

In summary, when CNTs undergo deformation, their C–C bond vibrations change, which alters the vibrational frequency of normal modes and causes Raman band changes. Therefore, Raman spectroscopy is a valuable tool to evaluate the quality and properties of carbon nanomaterials^22^.

### XRD spectrometry

**Figure 3:**
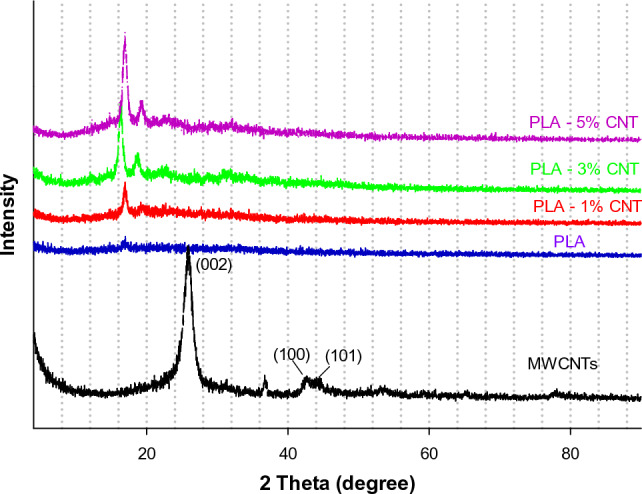
XRD patterns of PLA, MWCNTs, PLA with 1, 3, and 5% CNTs.

The X-ray diffraction (XRD) patterns of PLA, MWCNTs, PLA-1% CNTs, PLA-3% CNTs, and PLA-5% CNTs are presented in Fig. 3. The amorphous nature of PLA is revealed by a broad diffraction peak with a slight hump at 2θ = 16.86°. However, incorporating of CNTs resulted in sharp, intense peaks at 16.86° and broad peaks at 19.86°, indicating the promotion of PLA crystallization. Furthermore, the characteristic diffraction peaks for PLA crystallites (16.9, 19. and 24.1°) were observed, and their intensity increased with increasing CNT loading, implying an enhancement in the crystallization of PLA. Additionally, the XRD patterns of the PLA films showed two tiny diffraction peaks corresponding to both the α- and β-forms, which is consistent with previous studies^23^.

The XRD patterns of PLA and its nanocomposites containing different concentrations of MWCNTs exhibited nearly identical diffraction peaks, albeit with slight shifts, implying that the incorporation of MWCNTs did not alter the crystal structure of PLA. These shifts may be attributed to solvent-induced bonding interactions between PLA and MWCNTs^24^. The XRD analysis was conducted in the 2θ range of 5–80° with a scanning speed of 0.7°/s. The sharp peak at 2θ = 25.7° corresponds to the (002) plane of hexagonal graphite in MWCNTs. Also, there is a small diffraction peak at 2θ = 42.7°, corresponding to the (100) plane, which matches the carbon's crystal planes; those characteristic peaks in the XRD curve of MWCNTs disappeared from all the XRD curves of the synthesized nanocomposites. This observation suggests that the interlayer d-spacing of graphite increased due to the complete dispersion of MWCNTs in the PLA matrix^25,26^.

The presence of α-crystalline structures at 2θ = 16.6° and β-form at 2θ = 18.65° indicates the semi-crystalline nature of PLA. The diffraction intensity of the β-form increased substantially with increasing CNT loading, leading to a rise in the crystallinity of PLA. Furthermore, the incorporation of CNTs resulted in more crystalline phases of PLA.

### Contact angle

**Figure 4:**
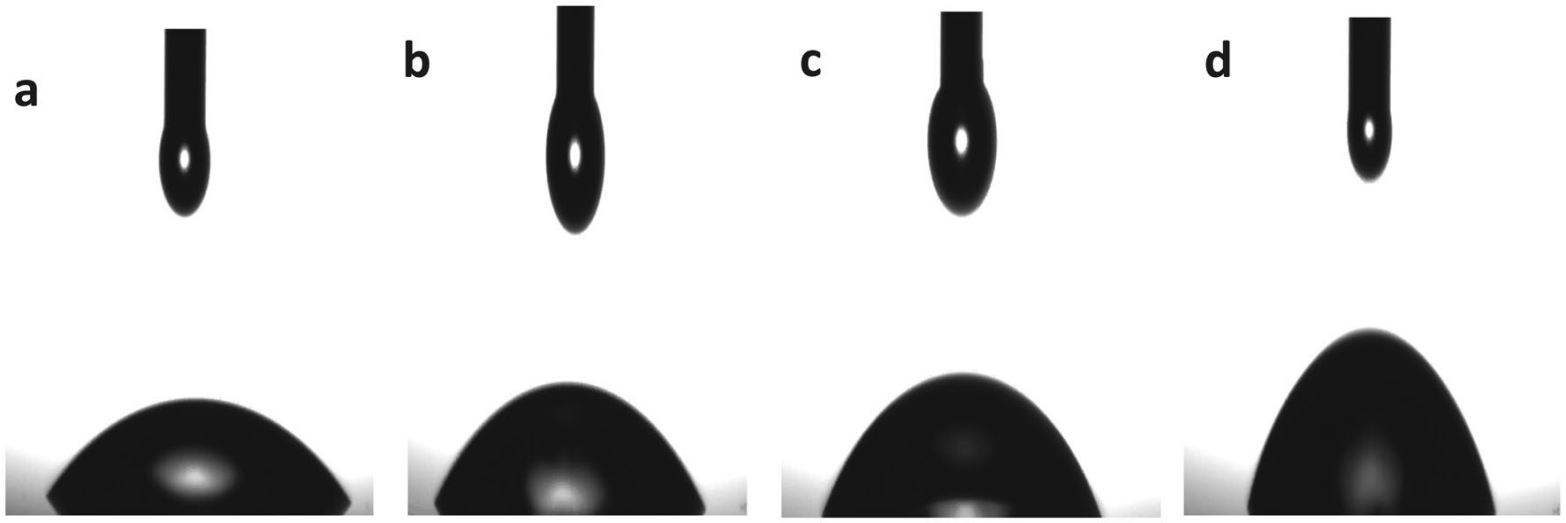
Contact angle of (**a**) PLA, (**b**) PLA-1% CNTs, (**c**) PLA-3% CNTs and (**d**) PLA-5% CNTs.

Contact angle measurement is a valuable technique for characterizing the surface wettability of materials. In this study, PLA and its nanocomposites with different concentrations of MWCNTs were subjected to contact angle measurement to investigate the effects of CNT loading on the wettability of the material. The contact angles of the samples were found to increase with increasing MWCNT content, indicating a decrease in surface hydrophilicity. The observed contact angles of the samples were 51°, 57.1°, 63.4°, and 72° for PLA, PLA-1% CNTs, PLA-3% CNTs, and PLA-5% CNTs, respectively, as shown in Fig. 4. The increase in contact angle with increasing CNT content suggests that the hydrophobicity of the composites is increasing with the addition of MWCNTs. The hydrophilicity of pure PLA is attributed to the presence of ester bonds, which make the surface more polar and, thus, more prone to water absorption. However, including hydrophobic MWCNTs in the PLA matrix results in a decrease in surface hydrophilicity, as evidenced by the increased contact angle. The decrease in surface hydrophilicity is expected to have important implications for the properties and applications of the composite material, as it can affect processes such as wetting and adhesion.

Overall, this study's results demonstrate that adding MWCNTs to PLA can significantly alter the surface wettability of the resulting composite material. Furthermore, the observed decrease in surface hydrophilicity with increasing CNT content is consistent with the hydrophobic nature of MWCNTs. It has important implications for the properties and applications of the material^27^. Further investigation is needed to fully understand the effects of CNT loading on the composite material's surface properties and optimize its performance for various applications.

### Transmission electron microscopy (TEM)

**Figure 5:**
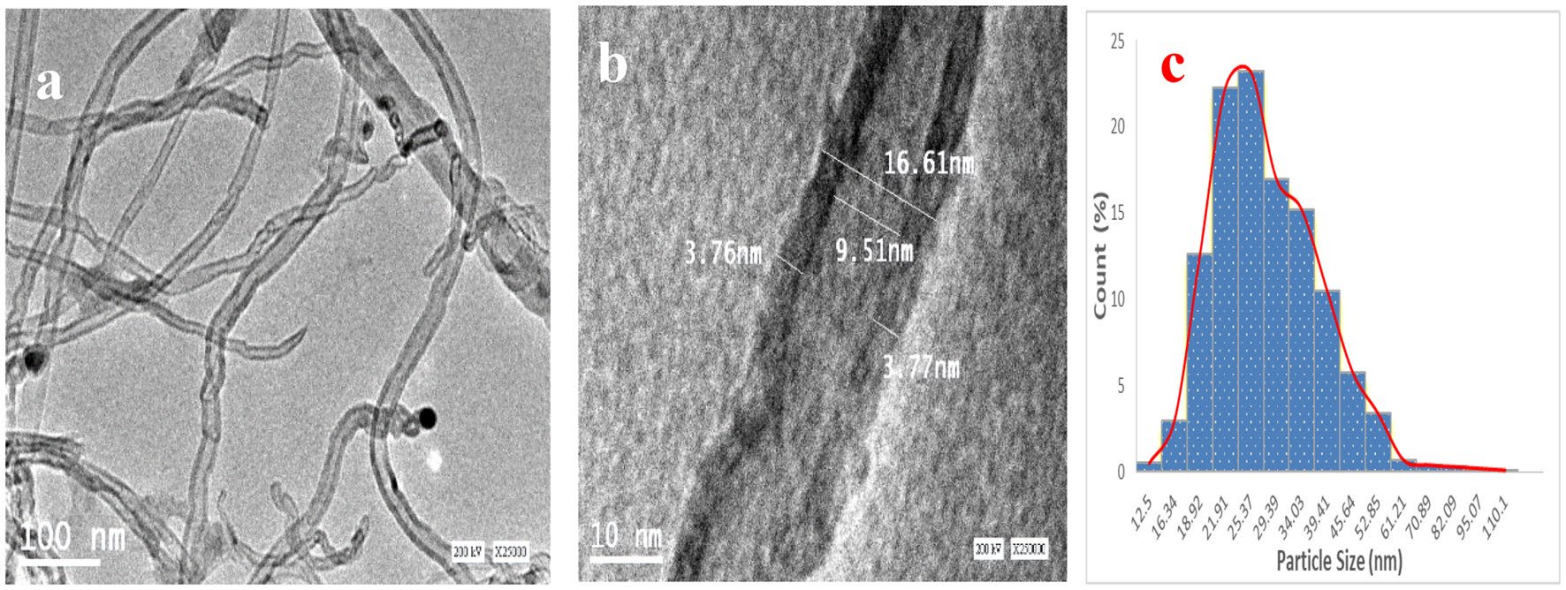
TEM image of MWCNTs (**a**,**b**) and their particle size distribution (**c**).

Transmission electron microscopy (TEM) is a powerful tool that provides high-resolution imaging and allows for detailed characterization of materials. The TEM analysis performed in this study revealed that the MWCNTs used in the preparation of composites had visible walls' uniform diameter distribution in the range of 17 nm, smooth surface, a high degree of entanglement, curved geometry, and cylindrical hollow holes^28^ as depicted in Fig. 5a,b. The particle size distribution of MWCNTs was between 10 and 40 nm, with an average diameter of 29 nm, as depicted in Fig. 5c, and the length was several microns. The absence of intrinsic flaws or oxidative damage in the MWCNTs confirmed their high quality, which is essential for obtaining composites with desirable properties. The particle size and morphology of MWCNTs are critical factors affecting the resulting composites' properties. The small size and high aspect ratio of MWCNTs promote better interfacial interactions with the polymer matrix, improving mechanical properties. Additionally, the uniform diameter and curved geometry of MWCNTs facilitate their dispersion in the polymer matrix, resulting in better homogeneity and improved properties. The absence of oxidative damage in MWCNTs is particularly important since it can impact composites' electrical conductivity and mechanical strength. Therefore, the high quality of MWCNTs used in this study could contribute to the excellent mechanical and electrical properties observed in the resulting composites.

Overall, the TEM analysis provides valuable information about the size, shape, and structure of MWCNTs, which are crucial factors for understanding the behavior of composites. The results of this study demonstrate the importance of using high-quality MWCNTs in preparing composites with desirable properties. Further research is needed to investigate the relationship between the particle size, morphology, and properties of MWCNTs and their composites.

### Field emission scanning electron microscope

**Figure 6:**
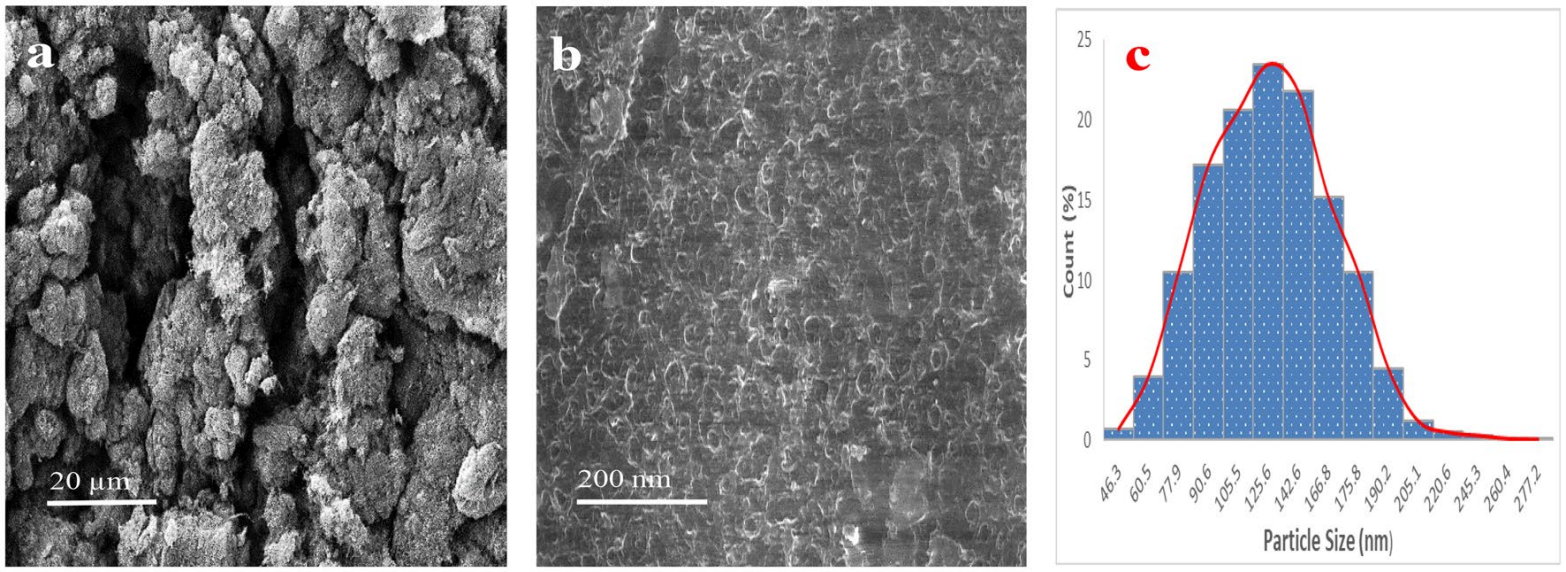
SEM images of PLA-3%CNTs composite (**a**,**b**) and their particle size distribution (**c**).

The use of advanced microscopy techniques is crucial in investigating the morphological structures of materials. In this work, we employed a scanning electron microscope (SEM) to examine the morphological features of the PLA-3%CNTs composite at different magnifications as shown in Fig. 6a,b. The primary objective of the analysis was to evaluate any changes that occurred due to the incorporation of CNTs into the PLA matrix. The SEM micrographs revealed a normal and smooth surface morphology of the PLA-3%CNTs composite, as shown in Fig. 6b, with high magnification, indicating that the material has a high degree of entanglement, is free of any noticeable flaws or cracks, and has random growth. The images also showed a well-distributed dispersion of CNTs within the PLA matrix^29^. This uniform distribution of CNTs can significantly impact the material's mechanical and electrical properties. Furthermore, a particle size distribution analysis was performed, which revealed an average particle size of 148 nm for the PLA-3%CNTs composite as shown in Fig. 6c. Interestingly, the addition of CNTs to the polymer particle increased particle size, indicating that the CNTs may have served as nucleation sites for particle growth.

The results obtained from the SEM analysis and particle size distribution provide valuable insights into the morphology of the PLA-3%CNTs composite. In addition, the uniform dispersion of CNTs and the increase in particle size demonstrate the potential benefits of incorporating CNTs into polymer matrices, such as improved mechanical and electrical properties. Overall, this study highlights the importance of advanced microscopy techniques in materials science research and development.

### Mechanical properties

**Figure 7:**
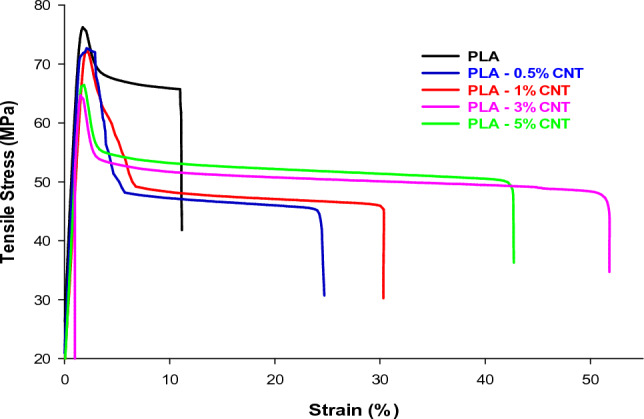
Tensile stress–strain curves of PLA and its nanocomposites with CNTs.

The tensile test was used to evaluate the mechanical performance of five different materials: PLA, PLA-0.5% CNTs, PLA-1% CNTs, PLA-3% CNTs, and PLA-5% CNTs. Figure 7 depicts our findings, which indicate that incorporating CNTs significantly improves PLA's toughness. The stress–strain curves showed that the elongation at break of pure PLA is limited, whereas the composites exhibit a significant elongation at break. Specifically, pure PLA, PLA-0.5% CNTs, PLA-1% CNTs, PLA-3% CNTs, and PLA-5% CNTs have an elongation at break values of 11, 24.4, 30.3, 51.8, and 42.7%, respectively, which are 1.2, 1.7, 3.7, and 2.8 times higher than that of pure PLA. Additionally, the ultimate tensile strength of the composites decreased compared to pure PLA. The ultimate tensile strength of pure PLA is 75.9 MPa, whereas that of the prepared composites is 72.2, 72, 64, and 66.2 MPa for PLA-0.5% CNTs, PLA-1% CNTs, PLA-3% CNTs, and PLA-5% CNTs, respectively. The E-modulus values decreased with the incorporating of CNTs; their values are 4.1, 3.9, 3.86, 3.3, and 3.19 GPa, respectively, as shown in Table 1. The addition of CNTs provides a strong reinforcement to PLA but also creates defects that reduce the ultimate tensile strength.

**Table 1:** The E-modulus, tensile strength, and elongation at break of pure PLA and PLA nanocomposites obtained from tensile tests.

Samples	E-modulus (GPa)	Max. tensile strength (MPa)	Elongation at break %
Pure PLA	4.1	75.9	11
PLA-0.5% CNTs	3.9	72.2	24.4
PLA-1% CNTs	3.86	72	30.3
PLA-3% CNTs	3.3	64	51.8
PLA-5% CNTs	3.19	66.2	42.7

To summarize, our results indicate that adding CNTs improves the toughness and elongation at the break of PLA. However, the ultimate tensile strength decreases as the loading of CNTs increases. These findings underscore the significance of the tensile test in evaluating the mechanical behavior of materials and offer insights into the potential applications of CNTs as reinforcing agents in polymer matrices.

### Dynamic mechanical analysis

**Figure 8:**
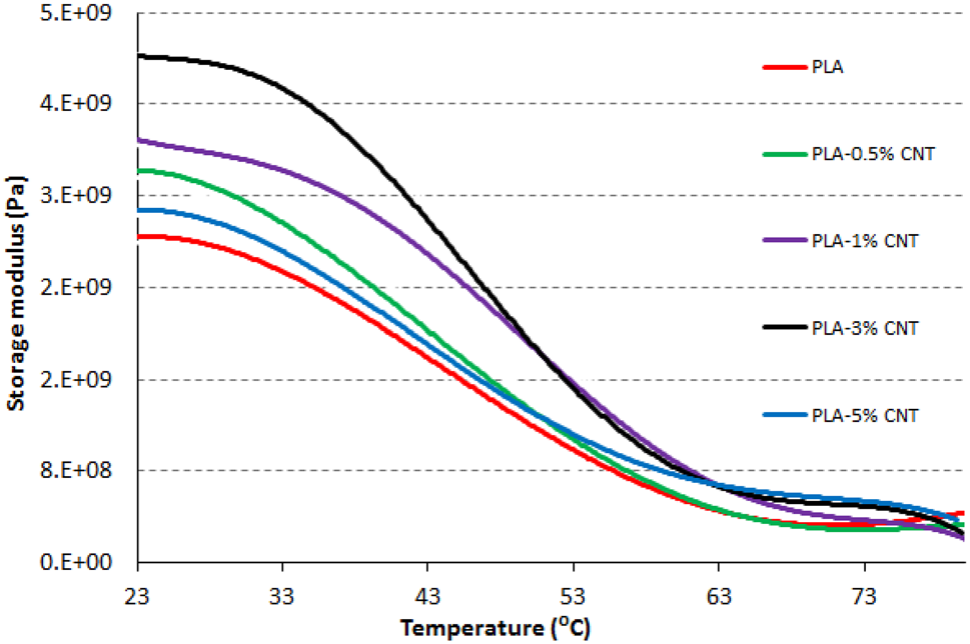
Modulus curves of PLA-CNT nonocomposites.

**Figure 9:**
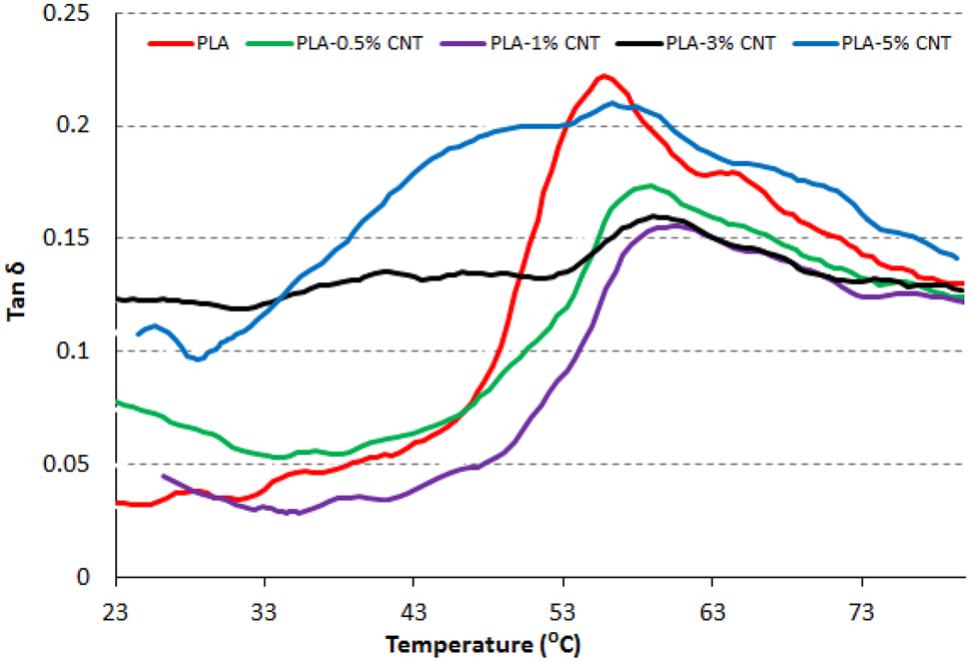
Tan δ curves of PLA-CNT nonocomposites.

The obtained data from DMA, in terms of storage modulus and tan δ of PLA-CNT nanocomposites, are illustrated in Figs. 8 and 9, respectively. The storage modulus curves in Fig. 8 exhibit comparable behavior in all nanocomposites and blank PLA, with two distinct phases. The first phase corresponds to the glassy phase and is present in the range of room temperature and 55 °C. This phase is characterized by controlled mobility of the polymeric segments and a sharp modulus decrease with temperature increase. The second phase appears after ~ 55 °C and corresponds to the rubbery phase, where the polymer has more free segmental motion. The transition between these two phases is visible in Fig. 9 as peaks in the obtained tan δ curves. It is reported that this transition between glassy and rubbery phases is known as the glass-transition temperature^30^. All nanocomposites exhibit improved moduli due to the effective CNT fillers. The blank, 0.5, 1, 3, and 5% PLA-CNT specimens recorded moduli of 2.6 × 10^9^ Pa, 3.2 × 10^9^ Pa, 3.4 × 10^9^ Pa, 4.2 × 10^9^ Pa, and 2.8 × 10^9^ Pa, respectively. The concentration of CNT filler significantly impacts the nanocomposites' modulus, with the highest modulus recorded for the 3% PLA-CNT nanocomposite. However, the 5% PLA-CNT nanocomposite exhibited a lower modulus than the other nanocomposites but was still higher than the blank. This decline in modulus may be attributed to the agglomeration of CNT. The highest peak in the tan δ curves was observed for the PLA polymer, and the addition of CNT lowered this peak due to the increased stiffness of the nanocomposites, resulting from restricted molecular mobility. This explanation is consistent with previous reports on decreasing tan δ peaks with increasing concentrations of nanofiller^31,32^. However, the tan δ peak of the 5% PLA-CNT nanocomposite increased again, which agrees with the modulus decline. Additionally, all nanocomposites showed broader peaks than blank PLA, which is affected by the restricted behavior of the reinforced polymer. The broadening of the tan δ curve is generally accompanied by progressive reinforcement^33^. Overall, the CNTs had a strong mechanical effect on the PLA matrix, particularly at the 3% concentration, resulting in a 61.5% increase in modulus compared to blank PLA and a lower and broader tan δ peak.

### Thermogravimetric analysis (TGA)

**Figure 10:**
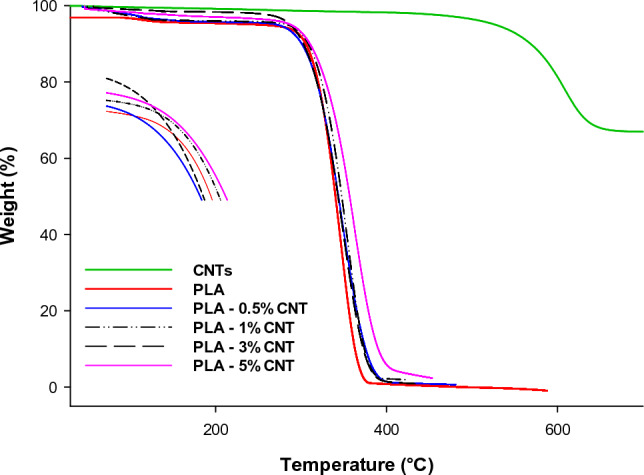
TGA curves of CNTs, PLA, and PLA/CNTs nanocomposites.

The thermal stability of a polymer is a crucial property in its processing and end-use applications. This study assessed thermal stability using thermogravimetric analysis (TGA) for pure PLA, CNTs, and PLA-CNT nanocomposites. Figure 10 shows the TGA curves, demonstrating that adding CNTs to PLA increased heat stability. This can be attributed to the physical barrier effect of CNTs and the restriction of polymer segment mobility^17^. Moreover, CNTs synthesized using Co/MgO catalysts were found to have better quality, higher graphitization, and fewer sidewall flaws, which resulted in more thermally stable carbon nanomaterials. Table 2 summarizes the thermal degradation data for pure PLA and PLA-CNT nanocomposites. The temperature at which 10 and 50% of the samples' weight were lost, and the residual weight at 450 °C were used for the analysis. The T_10_% and T_50_% breakdown temperatures were almost higher for all PLA/CNT composites than pure PLA. Specifically, the T_10_% decomposition temperatures for PLA, PLA-0.5% CNTs, PLA-1% CNTs, PLA-3% CNTs, and PLA-5% CNTs were 305, 300, 308, 304, and 309 °C, respectively. Meanwhile, these materials' T_50_% breakdown temperatures were 343, 348, 349.6, 352, and 362.06 °C, respectively. At 450 °C, the residual weight percentage was 0.1, 0.6, 0.97, 2.2, and 3.9%, respectively, notably, the char residue percentage increases with the percentage of CNTs, which naturally resist flames.

**Table 2:** Thermal properties of pure PLA and PLA nanocomposites.

Samples	Degradation temperature (°C)		Residual weight % At 450 °C
	T_10%_	T_50%_	
Pure PLA	305	343	0.1
PLA-0.5% CNTs	300	348	0.6
PLA-1% CNTs	308	349.6	0.97
PLA-3% CNTs	304	352	2.2
PLA-5% CNTs	309	362.06	3.9

Overall, the results suggest that including CNTs in PLA led to increased thermal stability, making the PLA/CNT composites more thermally stable than pure PLA. The data also indicate that the 5% PLA-CNT nanocomposite exhibited the highest thermal stability.

#### Degradation studies

**Figure 11:**
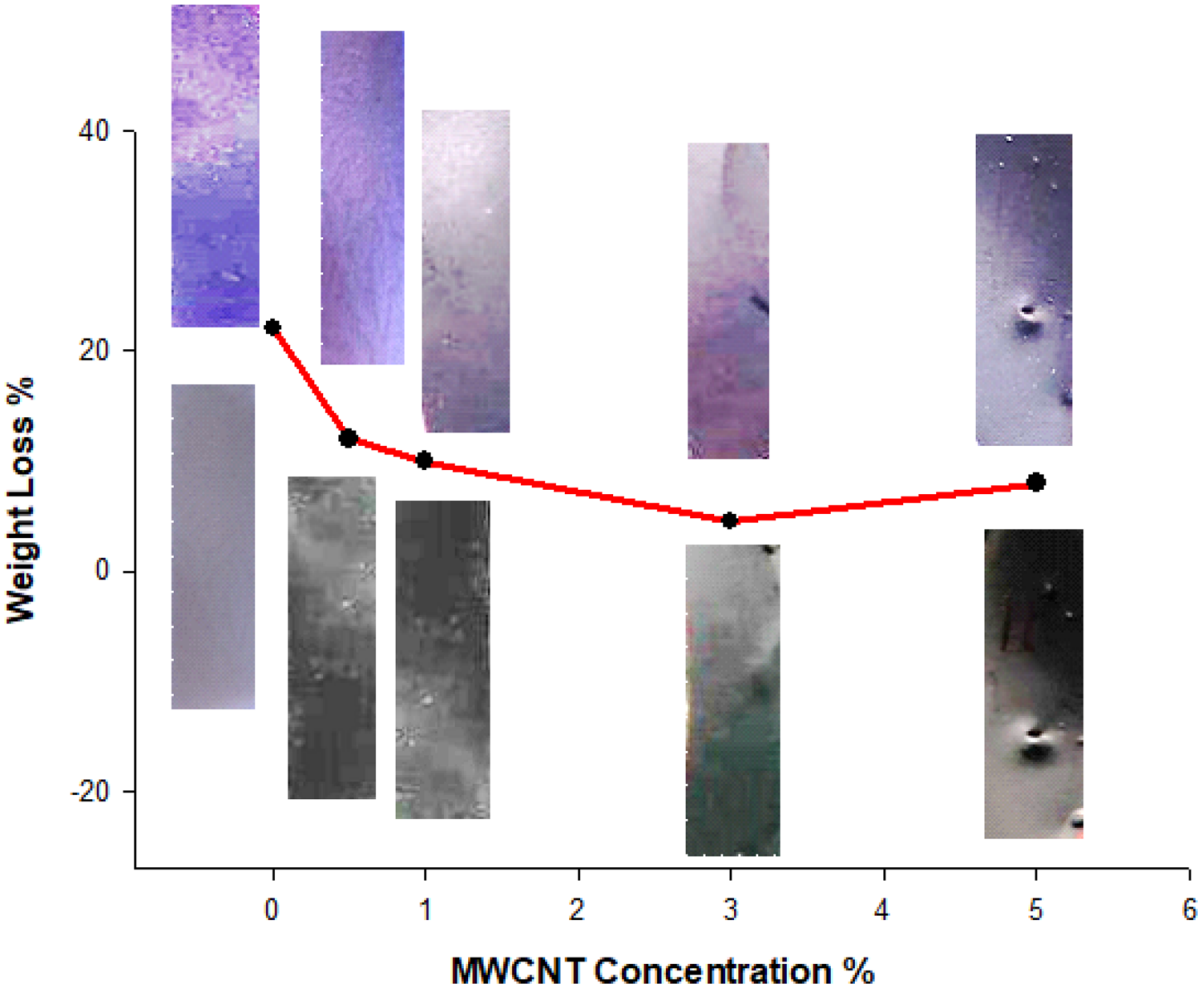
Weight loss curves of PLA and its nanocomposites with CNTs (The images positioned above the curve represent films subjected to weathering conditions, while the images positioned below the curve depict films that have not been exposed to weathering conditions).

Figure 11 represents the weight loss curves of PLA and its nanocomposites with CNTs as a function of CNTs concentrations. Adding multi-wall functionalized CNTs to PLA using the solution casting film technique in chloroform solvent can enhance the weathering resistance of the polymer. MWCNTs act as UV absorbers and scavengers and provide physical reinforcement. This can help prevent the formation of free radicals that initiate degradation reactions and maintain the integrity of the material during weathering exposure. The specific effect of different ratios of MWCNTs on PLA degradation depends on various factors such as the type and quality of CNTs, processing conditions, exposure time, and the nature of the weathering conditions. Our study exposed PLA films with different ratios of MWCNTs (0.5, 1, 3, and 5%) to activated weathering conditions, including UV, humidity, and 60 °C temperature, for 200 h. Our results showed that the addition of MWCNTs at a concentration of 3% resulted in the highest improvement in the weathering resistance of PLA only 4% of weight loss against 22% for neat PLA. This may be attributed to the optimal balance between physical reinforcement, UV absorption, and or scavenging provided by the MWCNTs at this concentration. Moreover, increasing MWCNT to 5% leads to less resistance. This can be explained by forming some agglomeration at this concentration, reducing the degradation resistance. However, further studies are necessary to determine the optimal concentration of MWCNTs for enhancing the weathering resistance of PLA under different weathering conditions.

The Images above and below the curve depict the pre- and post-weathering conditions of PLA and CNT nanocomposite films, as illustrated in Fig. 11. The pristine PLA image revealed the presence of numerous minute pores (surface roughness) after exposure to weathering conditions. Conversely, the images of the nanocomposites exhibited reduced roughness. The PLA matrix close to the surface underwent gradual degradation attributed to photodegradation. CNTs were utilized as heterogeneous nucleating agents, enhancing the surface area for polymer crystallization. In addition, incorporating CNTs facilitated a heightened nucleation density, preventing the formation of deterioration. Consequently, this discovery suggests that the CNT surface layer efficiently shielded the PLA matrix from deterioration^34^.

Overall, our findings suggest that incorporating MWCNTs into PLA can improve its weathering resistance, which may expand its potential applications in various industries, particularly those requiring long-term outdoor exposure^35^. However, additional research is needed to fully understand the effect of MWCNTs on the degradation of PLA and to optimize the processing and incorporation of MWCNTs into PLA matrices.

## Conclusion

In conclusion, the incorporation of multi-walled carbon nanotubes (MWCNTs) into polylactic acid (PLA) composites has been shown to have numerous benefits. Our experimental findings demonstrate that adding MWCNTs enhances PLA's thermal stability, making it more suitable for processing and end-use applications. We have also confirmed the good incorporation and dispersion of MWCNTs within the PLA matrix using FTIR and Raman spectroscopy. The XRD patterns indicate an increase in PLA crystallinity with the addition of CNTs. Furthermore, the hydrophobic properties of MWCNTs improve the contact angle of PLA and make it more resistant to UV degradation. The SEM micrographs reveal that the MWCNTs are well-distributed within the PLA matrix. The tensile test results demonstrate an increase in elongation at break, albeit with a slight decrease in ultimate tensile strength. The storage modulus values and tan δ of PLA-CNT nanocomposites were also improved compared to pure PLA, indicating a robust mechanical effect on the PLA matrix. Our study suggests that PLA-MWCNT nanocomposites have excellent potential in various applications, including packaging, medical devices, and textiles.

## Data Availability

All data supporting the findings of this study are available in the main text. The raw data used for the characterization of the prepared PLA/CNTs nanocomposite films, including FT-IR, Raman spectroscopy, TGA, TEM, SEM, and XRD results, are available upon request from the corresponding author. The CNTs prepared using catalytic chemical vapor deposition and the prepared PLA/CNTs nanocomposite films are available for academic research purposes from the authors. Any additional information related to the data or materials used in this study is available upon request from the corresponding author.
